# Breaking the Silence: A Scoping Literature Review on Trauma-Informed Care for Black Women Navigating Sexual Health-Related Trauma

**DOI:** 10.3390/ijerph22101484

**Published:** 2025-09-25

**Authors:** Ayanna Troutman, Funlola Are, Ashley Okoye, Sarah Chiang, Destiny Craig, Anthony Akande, Irene Stafford

**Affiliations:** 1 Louis A. Faillace, MD, Department of Psychiatry and Behavioral Sciences, McGovern Medical School, University of Texas Health Science Center at Houston, Houston, TX 77054, USAirene.stafford@uth.tmc.edu (I.S.); 2 Department of Psychiatry, Washington University School of Medicine, St. Louis, MO 63110, USA

**Keywords:** trauma-informed care, stigma, access to care, women’s health, mental health

## Abstract

Black women disproportionately experience sexual health-related trauma, yet their mental health needs are often inadequately addressed due to systemic barriers and stigma. This literature review examines trauma-informed care (TIC) frameworks tailored to Black women, emphasizing culturally responsive practices in addressing sexual health-related trauma. By synthesizing findings from existing research, including the importance of intersectionality, culturally specific interventions, and community-centered approaches, this review highlights effective strategies for mental health providers. The review concludes with implications for enhancing TIC training and implementation in clinical settings which contributes to the advancement of equitable mental health services for Black women.

## 1. Introduction

Trauma-informed care (TIC) is an important framework within mental health and healthcare systems [1]. Its goal is to recognize, understand, and respond to the widespread impact of trauma. Defined by the Substance Abuse and Mental Health Services Administration [2], TIC is based on six core principles: safety, trustworthiness and transparency, peer support, collaboration, empowerment, and cultural, historical, and gender responsiveness. TIC adopts a person-centered approach to understanding individual challenges rather than simply identifying what is wrong. The focus of this framework emphasizes the importance of recognizing how trauma shapes health outcomes, behaviors, and individuals’ engagement with healthcare systems and services.

Trauma is increasingly recognized as pervasive and interconnected with various social, structural, and cultural factors [3]. Consequently, TIC has been increasingly integrated into mental health interventions, policy recommendations, and service delivery models. However, despite this momentum, TIC is frequently inadequately applied across diverse populations, particularly among Black women [4]. In the United States, Black women experience disproportionate exposure to trauma, including sexual violence, intimate partner violence (IPV), and structural oppression stemming from racism, sexism, and economic inequality [5]. These intersecting forms of trauma uniquely affect their mental health, resulting in increased risks of depression, post-traumatic stress disorder (PTSD), substance use, and other health issues [6]. Yet, their access to culturally responsive, trauma-informed mental health care remains limited.

The gap in sexual health-related trauma is significant for Black women [7]. Historically, their sexual and reproductive autonomy has been controlled and neglected due to systemic policies and cultural narratives. To improve treatment outcomes, it is essential to address health disparities, including the overrepresentation of Black women among survivors of sexual violence and their limited access to mental health care. Often, services offered to these women do not affirm their identities or consider the sociopolitical factors impacting their mental health and healing [8].

Despite these interconnected challenges, TIC has the potential to be a powerful framework for healing when adapted with cultural responsiveness and equity in mind. A truly trauma-informed approach must move beyond generic principles and address the specific cultural, historical, and systemic contexts in which trauma occurs. For Black women, it is important to recognize the disproportionately high prevalence of trauma while also prioritizing their lived experiences, strengths, and community knowledge in the delivery of care. This approach requires rethinking TIC to incorporate culturally relevant definitions of healing. These definitions may include elements such as spirituality, collective care, and resistance to structural violence [9]. 

Importantly, TIC is most effective when its principles are applied to everyday practice in care delivery. This might include organizational changes such as staff training and treatment methods that emphasize choice, collaboration, and empowerment. In practice, strategies could include offering survivors’ options in their care, creating safe and transparent treatment environments, and incorporating peer or community-based support. These types of efforts can help build trust to support effective engagement. A more detailed discussion of the six core principles is provided in Section 2 of the manuscript.

Much of the existing literature on TIC does not specifically address Black women or adequately explore the ways in which race, gender, and structural inequities affect experiences of trauma and treatment outcomes. While there has been an increasing focus on TIC in schools, juvenile justice systems, and healthcare more broadly, there is still a limited synthesis of findings that specifically looks at trauma-informed mental health care for sexual health-related trauma among Black women. As a result, mental health professionals, researchers, and policymakers have little guidance on how to design or implement effective, culturally responsive TIC interventions for this population.

This scoping literature review focuses on the gap in research regarding trauma-informed mental health services for Black women who have experienced sexual health-related trauma. The review aims to identify key themes present in existing literature and assess the extent to which culturally relevant practices are incorporated into these services. A scoping review is the most appropriate methodology for this topic because the evidence base on TIC interventions for Black women survivors of sexual trauma remains limited and underexplored.

### 1.1. Contextualizing Sexual Health-Related Trauma Among Black Women

Sexual health-related trauma among Black women in the United States remains a critical public health issue that is consistently under addressed in the current literature despite noted health disparities [10]. To effectively understand Black women health, it is essential to understand these experiences through a historical and sociocultural lens that underscores bias in medical and legal systems, a history of systemic racism, sexism, as well as the intersection of the two, and cultural stigma [11]. As such, these often-intersecting factors shape the experiences of Black women who endure sexual trauma.

To ground this review in the lived realities of Black women, it is important to first contextualize the nature of sexual health-related trauma within this population. The following section outlines the historical, sociocultural, socio-economic, and systemic factors that contribute to both the prevalence of trauma and the barriers to effective care.

### 1.2. Sociocultural Barriers

Black women in the United States have long been subject to cultural stereotypes, many of which have been used to justify sexual violence against them or to excuse the lack of support they receive when such violence occurs [12]. One pervasive historical stereotype is the Jezebel, which portrays Black women as hypersexual and promiscuous [13,14]. Rooted in slavery, this stereotype was historically used to rationalize the sexual abuse of enslaved Black women by white men by framing them as willing participants, or even initiators, rather than victims of violence [15,16].

In contrast, the Mammy stereotype depicted Black women as undesirable caregivers, often responsible for the children of white families. This image portrayed Black women as incapable of experiencing abuse, not only because they were seen as undesirable, but also because they were viewed as the property of the families they cared for [17].

Another influential stereotype, the strong Black woman archetype, has contributed to the invalidation of Black women’s trauma experiences [18]. Although originally intended to celebrate resilience in the face of adversity, researchers have noted that this narrative can cause harm by suggesting that Black women should be capable of withstanding extreme abuse without assistance [19]. In the context of sexual trauma, this stereotype may invalidate a woman’s experience, implying she should possess a superhuman ability to prevent or fight off abuse, or, if it occurs, to simply carry on without support. Such assumptions negate Black women’s vulnerability and obscure their mental health needs, with help-seeking often viewed as a sign of weakness.

These and other harmful representations have contributed to society’s ongoing reluctance to acknowledge the sexual trauma Black women endure. As a result, Black women are frequently excluded or erased from broader conversations about sexual trauma and abuse. The lasting impact of these stereotypes continues to shape how Black women experience and disclose sexual violence, often resulting in disbelief, victim-blaming, or dismissal [20]. 

Stigma, both external and internalized, also plays a significant role in preventing Black women from accessing care and disclosing sexual trauma. Many studies have documented that fear of being blamed, judged, or not believed keeps many survivors of sexual violence from speaking out [21,22]. For Black women, this stigma is often heightened due to the intersection of racial and gender-based identities [23]. Additionally, cultural expectations around privacy and emphasis on keeping family and community matters private may lead to pressure to avoid bringing shame to one’s family by not disclosing or talking about abuse experiences, which often prolongs suffering and limits an individual’s ability to seek help and heal from trauma experiences [24]. 

### 1.3. Systemic Inequities Across Healthcare and Legal Sysems

Institutional racism within the healthcare system continues to pose a major barrier for Black women seeking treatment for sexual trauma. Disparities in access, quality of care, and provider bias all contribute to poor mental and physical health outcomes [25]. Compared to their white counterparts, Black women are less likely to receive appropriate mental health referrals, trauma-informed care, or consistent follow-up services [26]. Even when Black women do seek help, their symptoms are often misdiagnosed, dismissed, or minimized [27,28]. These challenges reinforce a longstanding mistrust of healthcare systems that stems not only from historical injustices but also from ongoing discriminatory practices and entrenched biases within the medical community [29]. The underrepresentation of Black providers in mental and sexual health fields further exacerbates the problem. While Black providers and clinicians may be better positioned to recognize and respond to culturally specific experiences of trauma, they are not free from systemic constraints or bias themselves making solutions to address these concerns particularly complex. Lastly, many healthcare settings lack adequate training in trauma-informed and culturally competent care, making it even more difficult for Black women to receive the support they need following sexual trauma experiences [30]. 

Beyond the healthcare system, Black women also face significant structural barriers in the criminal justice system when reporting sexual violence. Studies have shown that Black women are less likely than white women to be believed by law enforcement, less likely to have their cases investigated, and more likely to be criminalized themselves [31]. This is particularly true for women who are also marginalized by class, immigration status, or sexual orientation [32]. Moreover, Black women who are survivors of sexual trauma and who interact with the justice system, whether through child protective services, custody battles, or law enforcement, often face a punitive response despite being victims themselves [33,34]. The intersections of racism, sexism, and classism shape how their experiences are perceived and addressed to the disadvantage of Black women, particularly when compared to non-Black women’s experiences.

### 1.4. Socio-Economic Barriers

While structural and system barriers such as racism present many challenges additional socio-economic barriers such as unequal access to health care and chronic underfunding of community-based mental health services make it so that quality mental health care is often in settings that are difficult to reach [35]. As a result, Black women with limited economic resources are often forced to seek care in settings that are less likely to have quality services such as trauma-informed care. Additional economic barriers, including housing instability and food insecurity, can further deprioritize healthcare making it even less likely that high-quality, trauma-informed care will be available or utilized for women with few economic resources. To assess how well existing interventions serve Black women who have experienced sexual health-related trauma, it is essential to begin with a foundational understanding of TIC. The following section outlines the core principles of TIC and explains how each principle can be adapted to reflect the lived experiences of Black women.

## 2. TIC Framework

Understanding how TIC should function is essential before assessing how it has been applied to Black women’s sexual health-related trauma. The six core principles of TIC provide a foundation for evaluating whether existing interventions adequately address the unique cultural, historical, and gendered contexts shaping Black women’s experiences. These principles also serve as a benchmark for identifying gaps in current practices and opportunities for adaptation to ensure care is both trauma-informed and culturally responsive.

There are six core TIC principles that guide its practice and implementation: safety, ensuring that individuals are psychologically and physically safe; trustworthiness and transparency within the organization; peer support, which recognizes the value of shared experiences; collaboration and mutuality, emphasizing the importance of partnerships; empowerment, voice, and choice; and finally, recognition of cultural, historical, and gender issues [36]. When applied with cultural intentionality, these principles can be adapted to meet the specific needs of marginalized populations, including Black women.

### 2.1. Safety

Safety is a crucial principle that highlights the necessity for individuals to feel both psychologically and physically secure in healthcare settings. For black women, navigating the healthcare system can present significant challenges stemming from systemic oppression and a historical context of medical mistrust, such as the Tuskegee syphilis study which deceived and withheld treatment from African American men with syphilis [37] and the unethical use of Henrietta Lacks’ cells for research [38]. This history understandably contributes to skepticism toward healthcare, making it essential for providers to foster an environment that prioritizes safety through anti-oppressive narratives and proactive harm prevention [39,40]. 

### 2.2. Trustworthiness and Transparency

Trustworthiness and transparency are vital in promoting effective healthcare delivery. The legacy of historical injustices has created deep-seated distrust among African Americans. Therefore, healthcare providers must engage in honest and open communication to cultivate an atmosphere encouraging individuals to seek assistance without fear [41]. Providers can tailor their approach to build a more trusting therapeutic relationship by acknowledging the potential for mistrust. TIC approaches underscore the importance of transparency which is particularly important given that many Black women endorse lack of transparency during medical visits or failure of medical providers to adequately explain medical procedures [42]. When providers intentionally engage in activities such as providing time for building rapport with patients, engaging in warm verbal and nonverbal communication methods, including engaging in active listening and minimizing distractions such as computer screens, can help foster trust and transparency in the context of patient-provider relationships [43]. 

### 2.3. Peer Support

Peer support provides a valuable framework for creating networks where individuals can engage in shared experiences to foster mutual understanding and healing. For Black women, peer support can yield positive outcomes such as wisdom, mentorship, and strategies to combat inequality. These outcomes are key components for effectively adapting TIC to their specific needs [44].

### 2.4. Collaboration and Mutuality

Collaboration and mutuality emphasize the importance of honoring the preferences and wishes of individuals whenever possible. This is particularly significant for black women, whose voices and opinions are often marginalized. Culturally sensitive collaboration within TIC allows their needs and desires to be prioritized, leading to a more personalized and relevant care experience [45]. 

### 2.5. Empowerment, Voice, and Choice

This principle emphasizes the importance of giving individuals a sense of control over their healthcare. For Black women, feeling empowered in their choices is essential, and healthcare providers must approach this with sensitivity. Providers can present choices in a more structured way by offering two or three tailored options rather than an overwhelming list. Additionally, involving patients in shared decision-making, where they discuss the risks and benefits of treatment options with their provider, can enhance their sense of agency [46]. 

### 2.6. Recognition of Cultural, Historical, and Gender Issues

Recognizing cultural, historical, and gender issues is essential in developing an effective TIC framework, particularly for black women. Intersectionality plays a pivotal role in how they experience trauma and seek care [47]. Addressing the cultural context and socio-political history of individuals and their communities is vital for delivering trauma-informed care that is both gender-sensitive and culturally responsive [45,48]. This approach not only acknowledges the complexities of their experiences but also enhances the overall effectiveness of interventions.

By organizing the implementation of TIC around these six core principles, healthcare providers can better support black women and address the unique challenges they face in navigating healthcare systems.

## 3. Methods

This literature review was conducted in accordance with the PRISMA (Preferred Reporting Items for Systematic Reviews and Meta-Analyses) guidelines for scoping reviews. A review protocol was developed and registered with the Open Science Framework (OSF). The protocol can be accessed at https://osf.io/ (accessed on 26 May 2025). The OSF DOI is 10.17605/OSF.IO/2CYHJ. A PRISMA checklist was completed to ensure transparency and reporting of the review process.

We conducted a scoping literature review to identify empirical studies on TIC for Black women who have experienced sexual trauma. Searches were conducted across four major academic databases: PubMed, PsycINFO, Google Scholar, and JSTOR. Primary search terms included “Trauma-Informed Care” AND “Black women” AND “sexual trauma”. Alternative search terms were also used to broaden the scope and capture culturally relevant literature, such as “Culturally responsive mental health” AND “sexual health-related trauma”.

**Figure 1:**
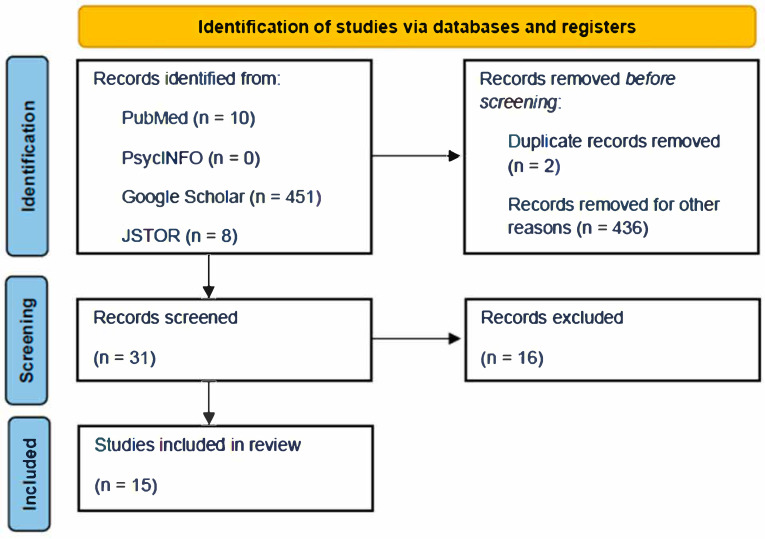
PRISMA Flow Diagram of Study Selection.

Studies were eligible for inclusion if they: (1) were published between 2015 and 2025, (2) were peer-reviewed empirical studies (i.e., qualitative, quantitative, or mixed-methods), (3) focused specifically on Black women, (4) addressed sexual trauma or sexual health-related trauma, and (5) incorporated or discussed TIC or similar frameworks. We excluded theoretical or conceptual papers without empirical data, studies not focused on Black women, and articles outside the specified date range. See Figure 1 for diagram of study selection. 

Titles and abstracts of N = 31 articles were screened by three authors to determine relevance. All potentially relevant articles were then reviewed in full by the first and second authors, with eligibility questions discussed and reconciled as a group. Additional studies were excluded if they did not focus on or include Black women in their sample and were outside of the specified timeframe. The final review included N = 15 studies. Ultimately, 16 studies were excluded from the 31 studies that were originally screened. Inter-coder agreement was established through independent review followed by discussion of discrepancies. Six articles required further discussion, and consensus was achieved through this process. Ultimately, these six articles were excluded because they did not adequately satisfy the coding criteria outlined because they were non- empirical papers (i.e., conceptual papers, book chapters).

Four researchers independently extracted data using a standardized form that included: (a) citation, (b) sample description (e.g., demographics, age), (c) study methods, (d) study setting, and (e) key findings. To ensure consistency, a study meeting was held to review extracted data and confirm accuracy. See Table 1 for more informations.

## 4. Literature Review Themes

### 4.1. Barriers to Accessing Mental Health Treatment

Despite growing momentum behind TIC frameworks, many Black women face significant barriers to engaging in mental health services, particularly following experiences of sexual health-related trauma. Cultural stigma, institutional mistrust, and economic strain are among the most pervasive barriers identified across literature. One of the most consistent findings is that stigmatizing attitudes around trauma and mental health reduces willingness to seek help. Xu et al. [61] found that trauma exposure among African American women was significantly associated with substance misuse, risky sexual behaviors, and poor mental health outcomes. However, stigma around mental illness and trauma often discouraged participants from accessing mental health support, even when symptoms were severe. Similarly, Samuel [56] explored Black women’s experiences of sexual trauma and healing and emphasized how cultural silence around sexual violence and expectations of strength contribute to internalized stigma and underutilization of services.

Institutional mistrust also plays a critical role. Ullman and Lorenz [60], in a large mixed-methods study of 836 African American sexual assault survivors, found that distrust of providers, fears of not being believed, and concerns about racial bias were major barriers to mental health help-seeking. Participants often shared experiences of feeling dismissed or misunderstood by their healthcare providers, which made them hesitant to seek care again. Supporting these findings, Willie et al. [36] interviewed both Black women and clinical staff and highlighted systemic provider bias as well as the need for culturally relevant care as critical issues in the implementation of trauma-informed PrEP, an HIV intervention. When healthcare systems do not create environments where Black women feel seen and respected, the trust essential for TIC is difficult to build.

These barriers are compounded by economic strain and structural limitations. Pegram and Abbey [54] compared African American and White women in suburban and semi-rural communities and found that African American survivors were more likely to experience chronic physical and psychological health effects following sexual trauma yet had less access to resources and supportive care. The authors emphasized that structural inequalities, including poverty, transportation issues, and lack of insurance, limit Black women’s ability to access consistent, trauma-informed services.

Sherman et al. [57] highlighted how systemic inequities manifest in clinical settings. Among 236 Black women recruited from STD clinics, the study found elevated mental health issues linked to sexual victimization. Even when women accessed health services, the care they received often did not meet trauma-informed standards. This indicates a disparity not only in access but also in the quality and responsiveness of care.

In a study conducted outside of the U.S., Amwiine et al. [49] analyzed the utilization of trauma-informed services among women in Uganda. They found that awareness and access to these services were low, primarily due to misinformation, stigma, and a lack of community-level awareness efforts. Although this research was carried out outside the U.S., it highlights the global presence of stigma and systemic barriers that impede access to TIC.

Community-based responses hold promise but need more investment. Decker et al. [31] found that many community organizations lack the resources to provide effective trauma-informed support for survivors. This underscores the need for improved culturally responsive training and infrastructure, especially in settings where Black women seek help.

These studies emphasize that stigma, mistrust, and systemic inequities are not just peripheral issues; they are central barriers that hinder Black women’s access to healing. For TIC frameworks to be effective, they must address not only individual trauma but also the structural and cultural conditions that impede Black women from receiving care in the first place. Future interventions should prioritize cultural humility, build community trust, and work to reduce material and systemic barriers to ensure that Black women can engage meaningfully in trauma-informed mental health services.

### 4.2. Culturally Responsive TIC

To be effective, TIC must move beyond generic frameworks and reflect the racial, gendered, and community-based experiences of those it aims to serve. For Black women, this means acknowledging how systemic oppression, community context, and lived experiences shape both trauma and healing. Culturally responsive TIC must be rooted in these realities to provide care that is equitable and relevant.

Several studies, presented in Table 1. highlight the importance of adapting TIC to account for these intersecting experiences. For example, Amwiine et al. [49], in a study conducted in Uganda, found that cultural and community-level barriers such as bribery, normalized beliefs around marital sexual violence, and family stigmatization discouraged women from seeking trauma-informed services. These findings emphasize the need for culturally grounded interventions that respond to the lived experiences of marginalized populations, including Black women globally.

Catabay et al. [50] also emphasized the value of culturally responsive care using the Minority Stress Model. In their study, Black women who experienced sexual violence reported high levels of perceived stress that were closely linked to symptoms of depression and anxiety. However, strong social support and personal resilience helped buffer these effects and were associated with better mental health outcomes. These findings highlight the importance of integrating protective cultural and social factors into TIC approaches.

Similarly, Sikkema et al. [58] implemented an intervention in South Africa for women living with HIV who had histories of sexual abuse. Participants in the culturally adapted ImpACT intervention reported reduced PTSD symptoms and increased motivation to adhere to antiretroviral therapy when compared to those receiving standard care. The use of culturally tailored materials demonstrated the importance of adapting TIC to align with participants’ real-life contexts.

In the United States, Chang et al. [51] explored how overlapping factors such as race, class, gender, and environmental stressors increase Black women’s vulnerability to HIV, particularly in low-income communities. This study incorporated open discussions about Black womanhood, encouraged the use of culturally specific language, and invited participants to reflect on their identity as part of the healing process. These components illustrate how culturally responsive interventions can enhance the impact of TIC when serving Black women.

Taken together, these studies affirm that the lived experiences of Black women are complex and shaped by intersecting systems of oppression. Culturally responsive TIC, when implemented with intentionality, can address not only the psychological and emotional effects of trauma but also the structural and cultural conditions that perpetuate harm. By centering these factors in intervention design and care delivery, providers can offer Black women access to care that supports healing, promotes well-being, and affirms their identities in systems that have too often failed to meet their needs.

### 4.3. Trauma Predicts Risk: Mental Health Distress, Substance Use, and Sexual Health Vulnerabilities

Another clear pattern across the literature reviewed is that trauma exposure is consistently associated with increased mental health concerns, substance use, and sexual health risks among Black women. While TIC provides a framework for recognizing and responding to trauma, the importance of its application is further underscored when considering the long-term consequences of unaddressed trauma. Trauma is not only a past event but an active influence on behaviors, relationships, and health outcomes. For Black women, these consequences are magnified by intersecting experiences of racism, gendered violence, and structural inequities.

Multiple studies affirm the relationship between trauma and heightened risk. Xu et al. [61] found that African American women with trauma histories were more likely to report depression, substance misuse, IPV, and sexual risk-taking behaviors. These findings demonstrate how trauma can create a cluster of interrelated health risks that unfold over time. This pattern aligns closely with Sherman et al. [57] who examined Black women recruited from STD clinics and found elevated mental health symptoms among those with histories of sexual victimization. These participants reported significant anxiety and depression, suggesting that even when women access healthcare services, the absence of TIC limits the system’s ability to provide meaningful support.

Pegram and Abbey [54] also highlighted how trauma severity among sexual assault survivors was associated with both psychological and physical health outcomes. Notably, Black women reported higher levels of chronic pain and distress compared to their White counterparts. This study overlaps with the theme of Barriers to Access, demonstrating that while trauma predicts risk, structural inequities, such as limited access to supportive care, further compound negative outcomes.

Samuel [56] contributes to this theme by exploring how trauma among many Black women often begins in childhood and continues across the lifespan. Many participants in the study described using avoidant coping strategies and noted a lack of safe spaces to process their trauma. This lack of support contributes to ongoing emotional distress and increases the risk of further harm. The study also emphasizes the impact of gendered racial microaggressions and the cultural silence surrounding trauma, which are interconnected with barriers to access and culturally responsive TIC. In environments that fail to affirm their identities and promote healing, Black women frequently carry the burden of trauma into adulthood, leading to behavioral and health risks.

Substance use is often discussed in the literature as both a risk factor and a coping mechanism. According to Xu et al. [61] individuals with trauma histories showed an increase in alcohol and drug use. Instead of viewing these behaviors as personal failures, TIC encourages providers to see substance use as a survival strategy that arises when support and care are lacking. This connection between trauma and maladaptive coping highlights the necessity for mental health services that prioritize harm reduction and culturally sensitive engagement, rather than adopting punitive or pathologizing approaches.

Findings from Stockman et al. [59] are also relevant to this theme. Their evaluation of the LinkPositively intervention, designed for Black women impacted by interpersonal violence, showed initial improvements in care engagement and treatment adherence. The feasibility of this technology-based trauma-informed care TIC model provides valuable insights into how targeted interventions can interrupt risk pathways by fostering consistent support and trauma-sensitive communication.

These studies collectively highlight that trauma is not just an isolated event; rather, it serves as a predictor of future vulnerability. The cumulative effects of trauma, especially in the absence of appropriate interventions, can lead to cycles of victimization, a decline in mental health, and reduced sexual and reproductive health. These outcomes further validate the need for widespread implementation of TIC across mental health and medical settings, particularly those serving Black women.

However, it is important to note that the scarcity of empirical studies specifically focused on Black women’s sexual health-related trauma is acknowledged as a limitation in capturing the full range of their experiences. Although relevant themes have been identified in the available literature, much of the research has been conducted on broader populations or contexts, which restricts the ability to draw strong conclusions tailored to Black women. Furthermore, the small pool of eligible studies limited opportunities for comparing outcomes across different trauma-informed interventions. These gaps are recognized as emphasizing the need for more focused and culturally responsive research in this area.

### 4.4. Implications for Practice and Research

This literature review emphasizes the urgent need to adapt TIC frameworks to better serve Black women who have experienced sexual health-related trauma. Although TIC provides a foundation for understanding and responding to trauma, its effectiveness is limited when interventions do not reflect the realities faced by those most affected. For Black women, these realities encompass the intersecting impacts of racism, sexism, classism, and historical marginalization. Addressing these complexities necessitates a shift in how TIC is conceptualized, implemented, and evaluated.

Culturally adapted TIC models are essential for providing meaningful support to Black women. It is important to understand trauma not only as an individual experience but also as a result of systemic harm. Culturally responsive TIC should incorporate the historical, cultural, and community contexts in which trauma occurs. The studies reviewed in this paper highlight that without considering cultural identity, lived experiences, and social location, trauma-informed interventions may become superficial and ineffective. The healing processes of Black women are often influenced by collective and ancestral knowledge, cultural traditions, and community-based coping mechanisms [62]. Effective TIC must reflect and honor these elements.

Protective factors such as social support, resilience, and culturally grounded coping strategies play a central role in the healing of Black women. Across multiple studies, women with access to affirming relationships, spiritual practices, and identity-based tools reported greater psychological well-being. TIC that integrates these protective factors can lead to improved engagement and outcomes. Rather than pathologizing how Black women cope with trauma, interventions should recognize and strengthen these strategies as essential sources of power and resistance.

Community-based settings can also provide promising access points for TIC. Trauma-informed services can be provided through trusted community institutions such as beauty salons, neighborhood clinics, peer-led support groups, and faith-based organizations, rather than relying solely on traditional mental health clinics [63]. These settings help reduce barriers to engagement by fostering familiarity, safety, and relational connections. Community-based interventions not only improve accessibility but also reinforce cultural affirmation, which is often lacking in formal systems.

Structural barriers such as poverty, inaccessible healthcare, and provider mistrust significantly limit access to care, particularly for marginalized populations. These systemic challenges must be directly addressed in the design and implementation of TIC frameworks. Economic instability, transportation issues, and negative experiences with healthcare providers also contribute to the underutilization of services [42]. Effective TIC requires not just comprehensive advocacy but also systemic reform, including policy changes aimed at expanding access, reducing costs, and creating safe treatment environments. Training healthcare providers in anti-oppressive practices is key for building trust and minimizing harm, particularly in the context of stigma, racial bias, and cultural ignorance that can lead to retraumatization.

Technology-based interventions present promising avenues for enhancing access to TIC. Tools like the LinkPositively app illustrate how mobile interventions can effectively support Black women survivors of IPV by providing psychoeducation, social support, and care engagement reminders in a flexible and private manner. When these digital platforms are developed with cultural responsiveness, they can bridge access gaps and work in harmony with in-person services. Future TIC interventions must prioritize gender-sensitive and racially responsive models that reflect the identities, histories, and healing practices of Black women. This requires integrating culturally specific language and ensuring therapeutic spaces and service delivery are affirming and adaptable.

There is a critical need for more research into the long-term outcomes of culturally responsive TIC. While existing studies indicate short-term improvements in trauma symptoms and care engagement, few have explored sustained impacts on areas such as substance use and quality of life. Future research should employ longitudinal designs and community-based participatory methods to yield relevant and actionable findings. Overall, the implications highlight that trauma-informed care should evolve to be deeply rooted in the experiences of Black women, rather than being considered a supplementary approach. It is essential for providers, researchers, and policymakers to work together to advance care models that address inequity, uplift community strengths, and affirm the dignity of marginalized populations.

## 5. Conclusions

This review emphasizes the urgent need for trauma-informed mental health services that are culturally relevant and responsive to the experiences of Black women affected by trauma related to sexual health. Across various studies, several consistent themes were revealed: trauma exposure is often exacerbated by systemic inequities, stigma, and a lack of culturally affirming care. TIC provides a critical foundation for addressing these challenges using its six principles of safety, trust, empowerment, collaboration, peer support, and sensitivity to cultural and historical factors. It offers a structured approach that can help providers recognize the impacts of trauma to work to prevent retraumatization. TIC has been widely adopted in healthcare and mental health settings because it encourages shifts in practice, policy, and culture. However, while TIC provides a useful framework, its effectiveness depends on how well it is adapted to reflect the intersecting racial, gender, and socioeconomic realities that shape the lives of Black women.

It is also important to acknowledge the limitations of this review’s methodological approach. The goal of the scoping review was to provide an overview of themes in the literature rather than evaluate study quality or a systematic synthesis of findings, such as a meta-analysis. This approach means the findings should be interpreted as suggestive rather than conclusive. The limited number of studies specifically focused on Black women also narrows the strengths of the conclusions and emphasizes the need for more rigorous and participatory TIC research.

Interventions that incorporate community voices, recognize structural harm, and utilize culturally rooted coping strategies have shown more promise than those that follow standard, one-size-fits-all models. To move forward, TIC must be reimagined in ways that honor the histories, healing traditions, and community strengths of Black women. It is essential for providers, researchers, and policymakers to ensure that TIC is implemented not merely as a checklist of principles, but as a practice grounded in justice and equity.

Future efforts should focus on the long-term evaluation of culturally responsive interventions, expand access through community-based and technology-enabled models, and address the structural conditions that continue to hinder Black women’s engagement in care. Prioritizing the voices of Black women in this work is crucial for establishing systems of care that genuinely support recovery and resilience.

## Figures and Tables

**Table 1:** Effective Interventions and Culturally Responsive Practices.

Author(s)	Origin	Study Type	Sample (N)	Methods	Setting	Summary
Amwiine et al. (2021) [49]	Uganda	Descriptive cross-sectional study qualitative design	32 females	Interviews	Kyangyenyi health center III and Kigarama health center III in Sheema district, southwestern Uganda	After assessing the knowledge of participants for trauma-informed services (TIS), the participants’ attitudes towards TIS, and different factors associated with the utilization of TIS, it was concluded that there was a knowledge gap about TIS and that sensitization needs to be done about this service
Catabay et al. (2019) [50]	USA	Quantitative, cross-sectional study	161 women	Self-report surveys	Community Based	Black women ages 18–44 with a history of sexual violence reported high levels of perceived stress, which were directly linked to symptoms of depression and anxiety. The presence of strong social support and personal resilience helped lessen the impact of stress on their mental health. Women with more support and higher resilience had better overall outcomes
Chang et al. (2023) [51]	USA	Mixed Methods	21 cisgender females	Quantitative surveys, Qualitative interviews	Community-based	Participants showed a decrease in trauma symptoms and increased confidence in managing their health and employment goals. Engagement in both HIV care and vocational services improved
Decker et al. (2025) [31]	USA	Qualitative	20 adults (15 Caucasian, 2 African American, 3 biracial, 2 AA/Caucasian, 1 Caucasian/Hispanic)	Semi-structured interviews	Recruited on social media (Facebook and Instagram) to take part in virtual interviews	Community-based organizations are important to address the health and support needs of survivors of sexual violence, and there are opportunities for areas of improvement in these organizations
Kaur et al. (2025) [52]	USA	Qualitative	12 Black women	Interviews	Community-based interviews	The study highlighted that childhood sexual abuse and intimate partner violence, significantly influences substance use behaviors among Black women. Additionally, social determinants of health, such as economic instability and access to healthcare, were found to play a crucial role in shaping health outcomes and coping mechanisms
Myers et al. (2019) [53]	South Africa	Mixed methods	60	Single-arm feasibility test and interviews via a group-based intervention	Community-based interviews	Participants reported reductions in substance use (methamphetamine, cannabis, and methaqualone), sexual risk behaviors (fewer sexual partners and less condomless sex), and symptoms of trauma-related mental health concerns, including depression, PTSD, and psychological distress
Pegram & Abbey (2019) [54]	USA (suburban and semi-rural communities in southeastern Michigan)	Quantitative study	221 participants (121 African American and 100 Caucasian women)	Standardized self-report measures	In-person interviews in various settings (e.g., participants’ homes, restaurants, coffee shops)	Examines racial differences in psychological and physical health outcomes among sexual assault survivors and highlights the need for racially responsive trauma-informed care
Ricks et al. (2023) [55]	USA	Mixed Methods	138 Black trans women	Interviews	In-person structured interviews	Black transgender women in the study reported high rates of childhood sexual abuse and intimate partner violence. Social gender affirmation was associated with improved mental health outcomes, but these benefits were weakened by recent or lifetime experiences of IPV
Samuel (2024) [56]	USA	Mixed methods	98 Black women	Rating scales and interviews	Online	Explores Black women’s experiences of sexual trauma and healing and emphasizes the role of racialized stressors and culturally specific coping strategies
Sherman et al. (2023) [57]	USA	Analyzation of cross-sectional data from a retrospective cohort study	236 Black women	Assisted self-interview	Recruited from STD clinics	Explores the exacerbation of mental health disorders associated with sexual victimization amongst black women
Sikkema et al. (2018) [58]	Cape Town, South Africa	Pilot clinical trial	64 women	Psychological intervention sessions	Primary health care clinic	Evaluated the feasibility of a coping intervention for HIV infected women with sexual abuse histories, preliminary findings show potential to reduce PTSD symptoms and increase ART adherence motivation
Stockman et al. (2023) [59]	USA	Pilot randomized control trial	80 Black women	LinkPositively intervention (mobile app)	In-person and online	Evaluated the feasibility of LinkPositively, a trauma-informed, tech-based HIV care intervention for Black women affected by interpersonal violence; found high acceptability and preliminary improvements in care engagement and treatment adherence
Ullman & Lorenz (2020) [60]	USA	Mixed methods	836 African American women	Standardized self-report measures and structured interviews	Surveys distributed via mail; interviews conducted in-person	Explored mental health help-seeking among African American sexual assault survivors, identifying key barriers including stigma, mistrust, and provider bias, and highlighting the importance of culturally sensitive care
Willie et al. (2023) [36]	USA	Qualitative	44 (37 Black women participants and 7 clinical staff members)	Semi-structured interviews	Community healthcare clinics	Identified key components for trauma-informed PrEP implementation based on perspectives from Black women and clinical staff in Mississippi; emphasized intimate partner violence (IPV) screening, staff training, and culturally relevant care
Xu et al. (2024) [61]	USA	Cross-sectional study	560 African American women	Standardized self-report measures administered via Audio Computer-Assisted Self-Interview (ACASI)	Community locations (e.g., beauty salons, shopping malls)	Assessed associations between trauma history and adverse psychosocial outcomes among young African American women; found that trauma was linked to higher substance misuse, risky sexual behavior, IPV, and poor mental health

## Data Availability

The data presented in this study are available on request from the first author.

## References

[1] Berring L.L., Holm T., Hansen J.P., Delcomyn C.L., Søndergaard R., Hvidhjelm J. (2024). Implementing trauma-informed care-settings, definitions, interventions, measures, and implementation across settings: A scoping review. Healthcare.

[2] Substance Abuse and Mental Health Services Administration Trauma-Informed Approaches and Programs. https://www.samhsa.gov/mental-health/trauma-violence/trauma-informed-approaches-programs.

[3] Raghavan S., Sandanapitchai P. (2024). The relationship between cultural variables and resilience to psychological trauma: A systematic review of the literature. Traumatology.

[4] Jieman A.T., Soliman F., York K., Bhui K., Onwumere J., Wynter S., Amasowomwan F., Johnson S., Jones J.M. (2024). Black women’s lived experiences of depression and related barriers and facilitators to utilising healthcare services: A systematic review and qualitative evidence synthesis co-produced with experts by lived experiences. medRxiv.

[5] Maldonado A.I., Murphy C.M., Davis M., Evans M.K., Zonderman A.B. (2022). Racial discrimination, mental health symptoms, and intimate partner violence perpetration in Black adults. J. Consult. Clin. Psychol..

[6] Lacey K., Parnell R., Mouzon D., Matusko N., Head D., Abelson J., Jackson J. (2015). The mental health of us black women: The roles of social context and severe intimate partner violence. BMJ Open.

[7] Eshelman L., Salim S., Bhuptani P., Saad M. (2023). Sexual objectification racial microaggressions amplify the positive relation between sexual assault and posttraumatic stress among black women. Psychol. Women Q..

[8] Matthews K., Morgan I., Davis K., Estriplet T., Perez S., Crear-Perry J. (2021). Pathways to equitable and antiracist maternal mental health care: Insights from black women stakeholders. Health Aff..

[9] McNeal R., Harris M., Oliphant V. (2024). Re-envisioning community-engaged healing for Black women. J. Int. Women’s Stud..

[10] Prather C., Fuller T.R., Jeffries W.L., Marshall K.J., Howell A.V., Belyue-Umole A., King W. (2018). Racism, African American women, and their sexual and reproductive health: A review of historical and contemporary evidence and implications for health equity. Health Equity.

[11] Chinn J.J., Martin I.K., Redmond N. (2021). Health equity among Black women in the United States. J. Women’s Health.

[12] Taft C.T., Bryant-Davis T., Woodward H.E., Tillman S., Torres S.E. (2009). Intimate partner violence against African American women: An examination of the socio-cultural context. Aggress. Violent Behav..

[13] Roberts D. (2010). The paradox of silence and display: Sexual violation of enslaved women and contemporary contradictions in Black female sexuality. Beyond Slavery: Overcoming Its Religious and Sexual Legacies.

[14] Cheeseborough T., Overstreet N., Ward L.M. (2020). Interpersonal sexual objectification, Jezebel stereotype endorsement, and justification of intimate partner violence toward women. Psychol. Women Q..

[15] Collins P.H. (2022). Black Feminist Thought: Knowledge, Consciousness, and the Politics of Empowerment.

[16] Melson-Silimon A., Spivey B.N., Skinner-Dorkenoo A.L. (2024). The construction of racial stereotypes and how they serve as racial propaganda. Soc. Personal. Psychol. Compass.

[17] Harris-Perry M.V. (2011). Sister Citizen: Shame, Stereotypes, and Black Women in America.

[18] Crooks N., Barrie R., Singer R., Donenberg G. (2023). The role of the strong black woman in Black female sexual development. Arch. Sex. Behav..

[19] Subhan B.A., Johnson V.E. (2023). The Strong Black Woman Archetype and therapeutic outcomes: Examining relationships among women with childhood sexual abuse histories. J. Racial Ethn. Health Disparities.

[20] Rosenthal L., Lobel M. (2016). Stereotypes of Black American women related to sexuality and motherhood. Psychol. Women Q..

[21] Kennedy A.C., Prock K.A. (2018). “I still feel like I am not normal”: A review of the role of stigma and stigmatization among female survivors of child sexual abuse, sexual assault, and intimate partner violence. Trauma Violence Abus..

[22] Tillman S., Bryant-Davis T., Smith K., Marks A. (2010). Shattering silence: Exploring barriers to disclosure for African American sexual assault survivors. Trauma Violence Abus..

[23] Gómez J.M., Gobin R.L. (2020). Black women and girls &# MeToo: Rape, cultural betrayal, & healing. Sex Roles.

[24] Bryant-Davis T., Ullman S.E., Tsong Y., Gobin R. (2011). Surviving the storm: The role of social support and religious coping in sexual assault recovery of African American women. Violence Against Women.

[25] Smedley B.D., Stith A.Y., Nelson A.R. (2003). Patient-provider communication: The effect of race and ethnicity on process and outcomes of healthcare. Unequal Treatment: Confronting Racial and Ethnic Disparities in Health Care.

[26] Powers A., Woods-Jaeger B., Stevens J.S., Bradley B., Patel M.B., Joyner A., Smith A.K., Jamieson D.J., Kaslow N., Michopoulos V. (2020). Trauma, psychiatric disorders, and treatment history among pregnant African American women. Psychol. Trauma Theory Res. Pract. Policy.

[27] Van Den Tillaart S., Kurtz D., Cash P. (2009). Powerlessness, marginalized identity, and silencing of health concerns: Voiced realities of women living with a mental health diagnosis. Int. J. Ment. Health Nurs..

[28] Wilson S.L. (2024). Understanding the hidden struggles: Cultural and somatic expressions of depression and anxiety in Black women. Women Health Care Issues.

[29] Whaley A.L. (2001). Cultural mistrust: An important psychological construct for diagnosis and treatment of African Americans. Prof. Psychol. Res. Pract..

[30] Reeves E. (2015). A synthesis of the literature on trauma-informed care. Issues Ment. Health Nurs..

[31] Decker H., Combs R., Lorenz K., Harris L., Wendel M. (2025). Reimagining support after sexual violence: Survivors’ recommendations for community-based support organizations. J. Soc. Serv. Res..

[32] Vohra-Gupta S., Petruzzi L., Jones C., Cubbin C. (2023). An intersectional approach to understanding barriers to healthcare for women. J. Community Health.

[33] Sharpless L., Kershaw T., Knight D., Campbell J.K., Phillips K., Katague M., Willie T.C. (2024). Moving towards transformative justice for black women survivors of intimate partner violence: An intersectional qualitative study. BMC Public Health.

[34] Slovinsky T.L. (2023). The thread of trauma: A critical analysis of the criminal legal system. Soc. Sci..

[35] Fischer M., Swint J., Zhang W., Zhang X. (2024). Mind the gap: Unraveling mental health disparities in America’s diverse landscape. medRxiv.

[36] Willie T.C., Phillips K., Shah A., Monger M.M., Nunn A., Kershaw T., Chan P.A., Baral S.D., Mayer K.H., Adimora A.A. (2023). Perspectives on HIV pre-exposure prophylaxis (PrEP) implementation in Mississippi among Black women and clinical staff: Recommendations for clinical trauma-informed programs. Prev. Med. Rep..

[37] Nnoli A. (2023). Historical primer on obstetrics and gynecology health inequities in America: A narrative review of four events. Obstet. Gynecol..

[38] Baptiste D.L., Caviness-Ashe N., Josiah N., Commodore-Mensah Y., Arscott J., Wilson P.R., Starks S. (2022). Henrietta Lacks and America’s dark history of research involving African Americans. Nurs. Open.

[39] Bloom S.L. (2013). Creating Sanctuary: Toward the Evolution of Sane Societies, Revised ed..

[40] Brown L.S. (2008). Cultural Competence in Trauma Therapy: Beyond the Flashback.

[41] Adams C.N., Hackman A.L. (2025). Trust and mistrust of mental health services among Black adults. Psychiatr. Serv..

[42] Washington A., Randall J. (2023). “We’re not taken seriously”: Describing the experiences of perceived discrimination in medical settings for black women. J. Racial Ethn. Health Disparities.

[43] Morera-Balaguer J., Botella-Rico J.M., Martínez-González M.C., Medina-Mirapeix F., Rodríguez-Nogueira Ó. (2018). Physical therapists’ perceptions and experiences about barriers and facilitators of therapeutic patient-centred relationships during outpatient rehabilitation: A qualitative study. Braz. J. Phys. Ther..

[44] Bryant-Davis T., Fasalojo B., Arounian A., Jackson K.L., Leithman E. (2021). Resist and rise: A trauma-informed womanist model for group therapy. Women Ther..

[45] Gutowski E.R., Badio K.S., Kaslow N.J. (2022). Trauma-informed inpatient care for marginalized women. Psychotherapy.

[46] Wilson J.M., Fauci J.E., Goodman L.A. (2015). Bringing trauma-informed practice to domestic violence programs: A qualitative analysis of current approaches. Am. J. Orthopsychiatry.

[47] Crenshaw K. (1991). Mapping the margins: Intersectionality, identity politics, and violence against women of color. Stanf. Law Rev..

[48] Comas-Díaz L., Alvarez A.N., Liang C.T.H., Neville H.A. (2016). Racial trauma recovery: A race-informed therapeutic approach to racial wounds. The Cost of Racism for People of Color: Contextualizing Experiences of Discrimination.

[49] Amwiine E., Ainembabazi B., Obwona I., Opoka R., Akatuhumuriza M., Niyonzima V., Mubangizi V. (2021). Perceptions of females about trauma-informed services for survivors of sexual violence in south western Uganda—A qualitative study. BMC Public Health.

[50] Catabay C.J., Stockman J.K., Campbell J.C., Tsuyuki K. (2019). Perceived stress and mental health: The mediating roles of social support and resilience among black women exposed to sexual violence. J. Affect. Disord..

[51] Chang H.Y., Johnson V., Conyers L.M. (2023). Exploring the impact of an integrated trauma-informed HIV and vocational intervention for Black/African American women living with HIV. Int. J. Environ. Res. Public Health.

[52] Kaur V., Lindinger-Sternart S., MacDonald D.A., Martin T. (2025). The interrelatedness of trauma, substance use, and the social determinants of health in Black women. Alcohol. Treat. Q..

[53] Myers B., Carney T., Browne F.A., Wechsberg W.M. (2019). A trauma-informed substance use and sexual risk reduction intervention for young South African women: A mixed-methods feasibility study. BMJ Open.

[54] Pegram S.E., Abbey A. (2019). Associations between sexual assault severity and psychological and physical health outcomes: Similarities and differences among African American and caucasian survivors. J. Interpers. Violence.

[55] Ricks J.M., Horan J. (2023). Associations between childhood sexual abuse, intimate partner violence trauma exposure, mental health, and social gender affirmation among Blacktransgender women. Health Equity.

[56] Samuel D.A. (2024). Ain’t I a Survivor Too: Contextualizing Black Women’s Experience of Sexual Trauma and Healing. Ph.D. Thesis.

[57] Sherman A.D.F., Cimino A.N., Balthazar M., Johnson K.B., Burns D.D., Verissimo A.D.O., Campbell J.C., Tsuyuki K., Stockman J.K. (2023). Discrimination, sexual violence, depression, post-traumatic stress disorder, and social support among Black women. J. Health Care Poor Underserved.

[58] Sikkema K.J., Mulawa M.I., Robertson C., Watt M.H., Ciya N., Stein D.J., Cherenack E.M., Choi K.W., Kombora M., Joska J.A. (2018). Improving AIDS Care After Trauma (ImpACT): Pilot outcomes of a coping intervention among HIV-infected women with sexual trauma in South Africa. AIDS Behav..

[59] Stockman J.K., Anderson K.M., DeSoto A.F., Campbell D.M., Tsuyuki K., Horvath K.J. (2023). A trauma-informed HIV intervention (LinkPositively) to improve HIV care among Black women affected by interpersonal violence: Protocol for a pilot randomized controlled trial. JMIR Res. Protoc..

[60] Ullman S.E., Lorenz K. (2020). African American sexual assault survivors and mental health help-seeking: A mixed methods study. Violence Against Women.

[61] Xu M.A., Choi J., Capasso A., DiClemente R. (2024). Association of trauma history with current psychosocial health outcomes of young African American women. Youth.

[62] Pettit-Toledo M.T. (2022). Collective memory and intersectional identities: Healing unique sexual violence harms against women of color past, present and future. Univ. Hawaiʻi Law Rev..

[63] Hong S. (2024). Trauma-informed cultural humility mental health practice: Centering history among African American women. Soc. Work.

